# Aquaporin locus (12q13.12) might contribute to susceptibility of temporomandibular joint disorder associated with periodontitis

**DOI:** 10.1371/journal.pone.0229245

**Published:** 2020-03-04

**Authors:** Mariana Bezamat, Emanuelle J. Cunha, Adriana M. Modesto, Alexandre R. Vieira, Juan M. Taboas

**Affiliations:** 1 Department of Oral Biology, University of Pittsburgh, Pittsburgh, Pennsylvania, United States of America; 2 Graduate Program of Dentistry, Pontífice Universidade Católica do Paraná, Curitiba, Paraná, Brazil; 3 Department of Pediatric Dentistry, University of Pittsburgh, Pittsburgh, Pennsylvania, United States of America; Ohio State University, UNITED STATES

## Abstract

Aquaporins (AQPs) are membrane channels that provide for transport of water and other small molecules across the lipid bilayer of cells. Their function is essential for physiologic processes such as cell volume regulation, chondrocyte hypertrophy during appendicular skeletal growth, water reabsorption in the kidney tubules, and water excretion by the salivary glands. The ten *AQP* isoforms show tissue specificity and are involved in different pathologies and inflammatory diseases. This study addresses the hypothesis that arthritis, periodontitis, and temporomandibular joint disorders (TMDs) can be influenced by variation in the *AQP* genes at 12q13.12 locus. Salivary samples of 688 individuals were obtained from the Dental Registry and DNA Repository project at the University of Pittsburgh. Ten polymorphisms in four *AQP* genes (*AQP1*, *2*, *5*, and *6*) were genotyped and correlated to disease status as reported by patients. Associations were found between the single nucleotide polymorphism (SNP) rs467323 in *AQP2* and TMD in both genotypic (p = 0.03) and recessive (p = 0.02) models, and between rs1996315 in *AQP6* and periodontitis (p = 0.05). Combined analysis of TMD and periodontitis showed an association with rs3741559 in *AQP2* (p = 0.02). When conducting haplotype analysis of rs467323 and rs10875989 in *AQP2*, the haplotype CT showed an association with the TMD phenotype (p = 0.007). Our results suggest that the aquaporin locus at 12q13.12 may contribute to the pathogenesis of inflammatory conditions such as periodontitis and TMD. Thus, oral and skeletal health are correlated and potential susceptibility screening strategies may be developed.

## Introduction

Aquaporins (AQP) are protein membrane channels expressed in numerous cell types such as endothelial and epithelial cells [1]. There are thirteen different isoforms with varying expression across cells and organs [2]. In addition to transporting water, they facilitate transport of small neutral-charge solutes, cell migration [3] and neuronal signaling [4]. Considering their expression by cells of the innate and adaptive immune system [5–7], roles in inflammation and inflammatory diseases have been elucidated, including regulation of cell volume [8] and migration of neutrophils, macrophages and T cells [9–11].

*AQPs* apparently play a role in periodontitis, one of the biggest threats to oral health and the major cause of tooth loss [12, 13]. Periodontitis is a chronic inflammatory disease identified by the destruction of the tooth-supporting tissues including periodontal-ligament, alveolar bone, and cementum. *Aqp3* deficiency is associated with greater mortality in a murine model of bacterial periodontitis [7]. Furthermore, *AQP1* expression is increased in gingival tissues of individuals who have periodontitis, and expression decreases in healed periodontal mucosa [14]. Anjomshoaa et al. reported associations between polymorphisms in *AQP5* and high caries experience in four different populations [15]. Because *AQP5* is expressed in serous acinar cells of salivary glands, polymorphisms may impact salivary flow. A decrease in the volume of saliva might increase the adherence of plaque to dental surfaces, and thereby caries and periodontitis incidence [16]. However, the best evidence for *AQP5* on dental caries susceptibility suggests a role during dental enamel formation [15,16].

Less studied are potential associations between *AQP* polymorphisms and inflammatory joint diseases, namely arthritis and temporomandibular joint disorders (TMDs). *AQPs* are expressed by chondrocytes and synoviocytes of appendicular and temporomandibular joints [17]. These cells respond to inflammatory cytokines and arthritis with changes in expression of *AQPs* [18–21]. Recently, conclusive evidence that *Aqp4* mediates rheumatoid arthritis severity was demonstrated in a rat model [22].

*AQPs* may be associated with both periodontitis and degenerative joint diseases because of the common inflammatory processes underlying these. In addition, periodontitis is associated with several other systemic inflammatory conditions [23–26], including rheumatoid arthritis [27]. We hypothesized that associations between *AQPs* and periodontitis, arthritis, and TMDs can be better detected when individuals concomitantly affected by these three conditions are studied. *AQP2*, *5* and *6* are clustered on the same locus, 12q13.12, while *AQP1* is the first identified constitute of the aquaporin family and the most studied *AQP* gene so far [28]. Therefore, we investigated the associations between periodontitis, arthritis, and TMDs and SNPs in the aquaporin locus at 12q13.12 and *AQP1*.

## Materials and methods

Individual samples and clinical history were obtained through the Dental Registry and DNA Repository of the School of Dental Medicine, University of Pittsburgh [29–31]. The total cohort consisted of 6,092 subjects with mean age of 59.1 years (ranging from 19.4 to 90.9 years-old). This project was approved by the University of Pittsburgh Institutional Review Board (IRB # 0606091). Written informed consent documents were obtained from all subjects. We followed the STREGA guidelines for this report.

Genotypes were generated blindly to clinical diagnosis status. Genomic DNA was extracted from whole saliva using established protocols [32]. Ten polymorphisms in four *AQP* genes (*AQP1*, *2*, *5*, and *6*) were analyzed (Table 1). PCR reactions were carried out using Taqman chemistry [33] in volumes of 3.0 μl in an ABI PRISM Sequence Detection System 7900 (Applied Biosystems, Foster City, CA, USA). The genotyping results were analyzed using SDS software version 1.7 (Applied Biosystems). PCR reactions were repeated twice when necessary. Genotypic (2df) tests were performed comparing genotypes to phenotype between affected individuals and their respective comparison group with PLINK software [34]. Additional haplotype analyses were performed utilizing Haploview software [35]. All 10 markers were consistent with Hardy-Weinberg equilibrium (Chi-squared p-values greater than 0.001) [34].

**Table 1:** Details of the SNPs investigated in this study.

Chromosome	Gene	SNP marker	Base Position	Location	Consequence	Base change
7	*AQP1*	rs17159702	30,919,387	intron	Intron Variant	CT
12	*AQP2*	rs467323	49,955,982	intron	3 Prime UTR Variant	CT
12	*AQP2*	rs3741559	49,951,193	intron	Intron Variant	AG
12	*AQP2*	rs461872	49,951,423	intron	Intron Variant	AG
12	*AQP2*	rs2878771	49,958,610	intron	3 Prime UTR Variant	CG
12	*AQP2*	rs10875989	49,957,292	intron	3 Prime UTR Variant	CT
12	*AQP5*	rs296763	49,969,231	intron	Non-Coding Transcript Variant	CG
12	*AQP5*	rs3759129	49,960,654	intron	2KB Upstream Variant	CA
12	*AQP5*	rs3736309	49,964,271	intron	Intron Variant	GA
12	*AQP6*	rs1996315	49,970,924	intron	2KB Upstream Variant	GA

### Sample and phenotypes selection

First, we selected an arthritis affected group, which consisted of individuals who self-reported osteoarthritis, rheumatoid arthritis or arthritis in our database. We selected 137 cases (47 males and 90 females). The mean age was 59.5 years, ranging from 19.4 to 90.9 years. For comparison we selected 551 unaffected individuals (191 males and 360 females), with a ratio of affected to unaffected of approximately 1:4 [36], matched to arthritis cases by age, ethnicity, and sex. Within the arthritis total sample, we selected the periodontitis, TMD groups, and unaffected groups using the same selection and matching criteria, except that periodontitis was not self-reported, but diagnosed by a professional dentist. For the periodontitis phenotype, individuals were considered affected if presenting with either at least three teeth exhibiting sites of clinical attachment loss and probing depth equal or greater to 5 mm in two different quadrants, or if presenting with alveolar bone resorption (evaluated radiographically) and severe dental mobility. A total of 141 affected individuals (93 females and 48 males) and 228 unaffected individuals (154 females and 74 males) constituted the periodontitis phenotype groups. For the TMD affected group, we selected 184 individuals (133 females and 51 males) that reported at least one symptom in the temporomandibular joint (clicking, sounds or pain) and 476 individuals (295 females and 181 males) constituted the comparison group. One of the authors (M.B.) carried out the extraction of clinical data after being calibrated by an experienced specialist (A.R.V.). The clinical data included the complete oral conditions and treatments present in the database for each of the patients seeking care between September 2006 and May 2018. The intra-extractor agreement was assessed by a second extraction of clinical data in 10% of the sample after 2 weeks, with a kappa of 1.0. Calculating inter- or intra-examiner agreement was not possible, because each phenotype studied was recovered from a registry of clinical information derived from the dental clinics of the University of Pittsburgh.

We estimated power for the association study by using the Genetic Power Calculator tool [37], setting the frequency of the high-risk allele in the population equal to 0.2, the prevalence of the conditions as 0.1, the effect size in the heterozygote form as 2.0, D’ as 1.0 (amount of linkage disequilibrium), the respective number of cases of each disease and the case-control ratio. The samples selected for arthritis, periodontitis, and TMD would have 76%, 62%, and 81% power, respectively, to detect association at an alpha of 0.05. We did not perform power calculations on the cohorts of individuals with more than one condition. One can assume that these subgroups of combined individuals with more than one disease would provide less statistical power because they are smaller, although we hope that this approach increases homogeneity.

### Linkage disequilibrium of the region and multiple testing concerns

**Fig 1:**
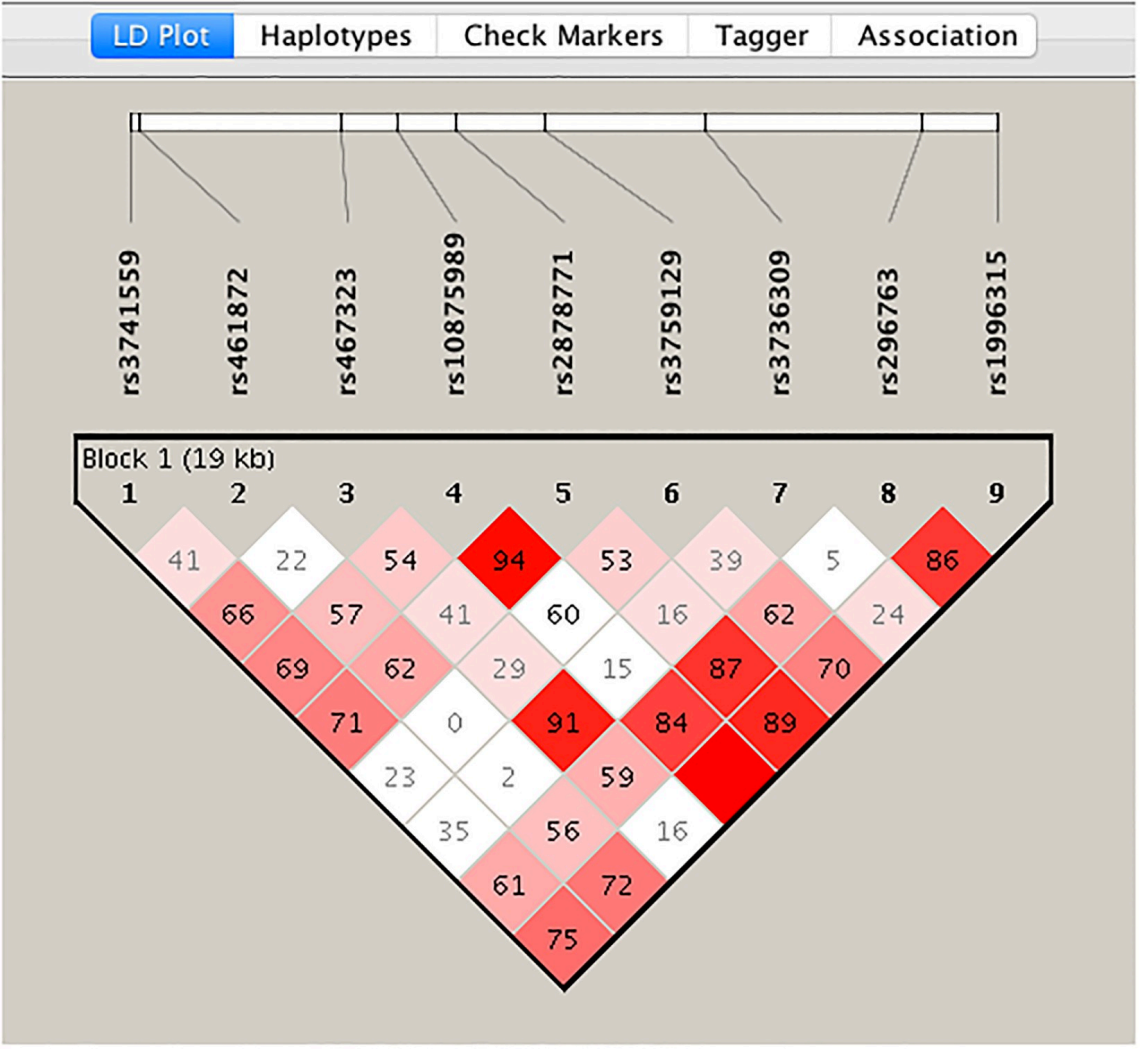
Illustrates the *AQP 2*, *5* and *6* markers of the 12q13.12 aquaporin locus. The different color intensities represent a different D prime value, the stronger the red, the closer a D’ value is to 1.

We generated a linkage disequilibrium plot of the 12q13.12 locus in this study population (Fig 1). We can conclude that the SNP rs467323 is in linkage disequilibrium with rs3736309, as well as the SNPs rs10875989, rs2878771, rs296763 and rs1996315, which mean these SNPs provide redundant information. We employed a Bonferroni correction for multiple comparisons, and set alpha at 0.01 (0.05/5 markers) to account for disequilibrium, excluding redundant information given by 4 SNPs.

## Results

P-values below 0.05 of periodontitis and temporomandibular joint symptoms were found and are represented in Table 2. The affected and unaffected individuals on the genotypic model represent the number of different genotypes (e.g. CC, CT and TT) in the affected group and in the unaffected group where the first number is the homozygous for the mutated alleles, followed by the heterozygous, followed by the homozygous for the normal alleles. We used our genotyping data to recalculate power since we could derive relative risks for AA, Aa and aa directly from our results. These post-hoc calculations showed our power for the comparisons of the sample selected for arthritis, periodontitis, TMD, and TMD combined with periodontitis were 12%, 58%, 83.5%, and 68.6%, respectively.

**Table 2:** Summarizes the nominal results of both phenotypic isolated and combined analyses.

Phenotype	A1 / A2	Gene	SNP	Model	Affected	Unaffected	X^2^	p value	Relative risk of carrying one copy of the risk allele
Periodontitis	G / A	*AQP6*	rs1996315	Dominant	106/23	150/55	3.583	0.058	1.2
Temporomandibular joint disorder (TMD)	C / T	*AQP2*	rs467323	Genotypic	18/84/56	80/178/149	6.706	0.03	1.46
		*AQP2*	rs467323	Recessive	18/140	80/327	5.421	0.02	
Periodontitis and TMD	A / G	*AQP2*	rs3741559	Allelic	25/177	45/171	5.355	0.02	2.15

### Single disease associations

We found an association between one marker (rs1996315) in *AQP6* and periodontitis (p = 0.05). Temporomandibular joint dysfunction was associated with one marker (rs467323) in *AQP2* for both genotypic (p = 0.03) and recessive (p = 0.02) models (Table 2). No association was found for arthritis with the markers tested.

### Dual disease associations

When individuals who reported both TMD and arthritis were analyzed, there were no statistically significant differences. However, when TMD and periodontitis were combined, a marker in *AQP2* (rs3741559) showed and association (p = 0.02) (Table 2).

### Triple disease associations

When the three disease phenotypes were combined (temporomandibular joint dysfunction, periodontitis, and arthritis), no associations were found.

### Haplotype analysis

We performed association tests of two or more adjacent SNPs within the 12q13.12 locus. The haplotype analysis revealed an additional association not previously identified by the single marker analysis. For rs10875989 and rs467323 in *AQP2*, the first SNP did not show signals alone but when combined with the second SNP, the haplotype CT showed a significant association with the TMD phenotype (p = 0.007), being approximately three times more frequently found in the control group (Table 3).

**Table 3:** Summarizes the haplotype significant association identified between rs467323 and rs10875989 block markers using the haploview software.

Haplotype Associations rs467323/rs10875989 block markers	Frequency	Case, Control Ratios	Chi Square	p value
TT	0.639	172.1: 91.9, 424.9: 245.1	0.257	0.6122
CC	0.283	77.5: 186.5, 187.1: 482.9	0.194	0.6596
CT	0.051	5.4: 258.6, 42.6: 627.4	7.212	**0.0072**
TC	0.026	8.9: 255.1, 15.4: 654.6	0.9	0.3428

## Discussion

*AQPs* have been reported to play a role in progression and resolution of inflammatory status [38]. Thus, here we analyzed inflammatory conditions that have different causes and chronic inflammation in common. We show that variation in *AQP2* are potentially associated with temporomandibular joint disorder and periodontitis. Our previous studies have shown that a more unique population is generated when disease phenotypes are analyzed in combination, increasing the chance of identifying biologically relevant associations [39]. Our intention in combining those diseases was to verify if a pleiotropic effect is present that leads those individuals to develop different conditions and symptoms at the same time. An association was found when patients who have both TMD and periodontitis were analyzed however, when all three diseases were analyzed in combination in this study, we did not find significant results. These negative results are likely due to the small sample size of patients affected by the three conditions concomitantly (n = 9).

Even though all markers complied with Hardy Weinberg equilibrium (HWE) cutoff of p>0.001 set as default in the PLINK software [34], the initial sample selected to define a cohort of individuals with arthritis had the genotype distribution of markers rs10875989 and rs2878771 different than expected under HWE (p = 0.009 and 0.004, respectively). It has been suggested [40] that this might happen due to population stratification, when both Whites and African-Americans are included in the study. Concerned with this possible effect we ran a separate analysis excluding African-Americans and we did not see a significant difference in HWE just for Whites [rs10875989, 25CC, 225CT and 207TT for Whites (p = 0.001) versus 44CC, 278CT and 257TT for the total sample (p = 0.009)] and [rs2878771, 6CC, 166CG and 295GG (p = 0.002) versus 12CC, 211CG and 378GG (p = 0.004)]. This indicates that any possible population substructure may be due to combinations of different groups of Europeans that cannot be detected with the information available to us [41]. The aquaporin locus in particular may make these individuals more susceptible to the inflammatory conditions we are studying, in comparison to African-Americans. The other possibility is that the selection of individuals with arthritis and the subsequent matched group (by sex, age, and ethnicity) may have caused this effect because these groups have an excess of heterozygous and homozygous rare individuals. This may be the reflex of the actual effect of the aquaporin locus on the underlying conditions studied since all sample is enriched by individuals affected by TMD and periodontitis. This effect was seen before when families with a child born with cleft lip and palate were analyzed based on paternal origin and showed Hardy-Weinberg distortion [42]. This effect would not necessarily translate to significant results detected by association methods because the overall population studied is enriched by the rare allele.

When sorting the subjects who were affected by more than one disease, it was interesting to observe that most of the patients who have periodontitis are also affected by TMD (106 individuals). This phenotype-to-phenotype finding correlates with the genotypic association results reported here. A recent study analyzed disease patterns of patients with temporomandibular disorders and concluded that chronic periodontitis resulting in unilateral mastication induces temporomandibular joint changes and pain is those patients [43]. Our study showed that a genetic component might also be present, increasing the chances of having temporomandibular joint disorders associated with periodontitis.

The rs1996315 marker is located between *AQP5* and *6* and is a substitution of G for A, being reported as associated with dental caries [44]. The suggested underlying mechanism of this association with caries might be due to reduced salivary flow, and likewise could affect dental plaque retention and increase the risk for periodontitis. The *AQP2* rs467323 marker is a substitution of C for T and the clinical significance is reported as benign, which means that the variant has no deleterious effect on gene expression or function [45]. Thus, our results demonstrate for the first time a relationship and possible protective effect of *AQP2* and TMD—the CC genotype is less seen in the affected group compared to the unaffected (11% and 19% respectively for rs467323 marker). The rs3741559 *AQP2* marker did not show associations with any of the diseases isolated but when individuals affected by both TMD and periodontitis were tested, the A allele was less present in the cases, characterizing a protective effect.

A limitation we faced in our study was that arthritis was self-reported by the patients; we did not always have a detailed description of the type of arthritis affliction. In our data, the results did not differ when rheumatoid arthritis and osteoarthritis patients are analyzed in separate and we decided to study both types together. Since we did not have an independent sample to replicate these results, independent investigations testing the aquaporin locus studied here in the inflammatory conditions we studied are warranted.

This study did not investigate all *AQPs* expressed by leukocytes and in the periodontium and cartilaginous tissues (e.g. *AQP 4*, *9*). Further work is needed to delineate if the effects of *AQP* polymorphisms in periodontitis and degenerative joint diseases are tissue specific (e.g. changes in epithelial barrier function) or immune response specific (e.g. phagocytosis), and to define their contribution to the type and severity of immune response to tissue injury and infection.

Periodontitis has been associated with a number of conditions that include cardiovascular risks and stroke, arthritis, lupus erythematosus, infections of the upper respiratory tract, diabetes, low birth weight and premature births (23–27). Here we provided statistical evidence that the aquaporin locus may be implicated in individuals affected with TMD and a trend to associations in individuals that show concomitant TMD and periodontitis, suggesting that this may be the reason why conditions that are inflammatory in nature are found to be associated with periodontitis in human populations.

## Supporting information

S1 Table Genotyping data used for analysis of arthritis group. (XLSX)

S2 Table Genotyping data used for analysis of periodontitis group. (XLSX)

S3 Table Genotyping data used for analysis of temporomandibular joint disorder group. (XLSX)

S4 Table Genotyping data used for analysis of arthritis and periodontitis combined group. (XLSX)

S5 Table Genotyping data used for analysis of arthritis and TMD combined group. (XLSX)

S6 Table Genotyping data used for analysis of periodontitis and TMD combined group. (XLSX)

S7 Table Genotyping data used for analysis of arthritis, periodontitis and TMD combined group. (XLSX)
